# *HvbZIP21*, a Novel Transcription Factor From Wild Barley Confers Drought Tolerance by Modulating ROS Scavenging

**DOI:** 10.3389/fpls.2022.878459

**Published:** 2022-04-22

**Authors:** Rui Pan, Sebastian Buitrago, Zhenbao Feng, Salah Fatouh Abou-Elwafa, Le Xu, Chengdao Li, Wenying Zhang

**Affiliations:** ^1^Research Center of Crop Stresses Resistance Technologies/Hubei Collaborative Innovation Center for Grain Industry, Yangtze University, Jingzhou, China; ^2^Department of Agronomy, Faculty of Agriculture, Assiut University, Assiut, Egypt; ^3^Western Crop Genetics Alliance, Western Australian State Agricultural Biotechnology Centre, College of Science, Health, Engineering and Education, Murdoch University, Murdoch, WA, Australia

**Keywords:** *Hordeum spontaneum*, water deficit, basic leucine zipper, reactive oxygen species, virus-induced gene silencing

## Abstract

Drought stress is a common environmental stress, which adversely affects the yield and quality of crops. Due to its excellent drought tolerance, wild barley from the Middle East region is considered a valuable source for barley improvement. Here, we compared the growth rate, stomatal regulation and capacity to metabolize reactive oxygen species (ROS) of two barley cultivars and one wild barley accession. The results indicated the wild barley EC_S1 showed a more significant decline in stomatal aperture and less ROS production. Transcriptomic analysis revealed that EC_S1 has slower transcriptional regulation (5,050 DEGs) in the early stage of drought stress (14 days) than Baudin (7,022 DEGs) and Tadmor (6,090 DEGs). In addition, 30 hub genes, including nine known drought-related genes were identified by WGCNA analysis. Then, we cloned a novel *bZIP* transcription factor, *HvbZIP21*, from EC_S1. *HvbZIP21* was subcellularly targeted to the nucleus. Overexpression of *HvbZIP21* in *Arabidopsis* enhanced drought tolerance due to increasing activities of superoxide dismutase, peroxidase, and catalase activities as well as glutathione content. Silencing of *HvbZIP21* in EC_S1 suppressed drought tolerance in BSMV:HvbZIP21-inoculated plants. Taken together, our findings suggest that *HvbZIP21* play a critical role in drought tolerance by manipulating ROS scavenging.

## Introduction

Water is the most essential condition for living matter. It is the medium, raw material, and place of life activities implicated in the two most important physiological processes: photosynthesis and transpiration. Drought induces several physiological changes in plants that can result in permanent wilting. In recent years, drought has intensified across the globe due to climate change, threatening crop productivity and potentially affecting economic stability and food security in vulnerable countries (Abou-Elwafa and Shehzad, 2021).

The effect of water deficit on plants involves almost all physiological and biochemical activities. Water moves through plants by the cohesion-tension created by vapor pressure demand (Kholová et al., 2012; Belko et al., 2013). When the available water in the soil is reduced, the transport chain is interrupted, triggering several signals. These signaling pathways, broadly classified into hydraulic and chemical signals, can be initiated by a high vapor pressure deficit within the shoots. Here, stomatal conductance and photosynthesis are hindered since the roots start abscisic acid (ABA) production, transporting it through xylem vessels to induce stomatal closure. As a result, the transpiration rate and photosynthesis are diminished. Under drought conditions, leaf water content is also affected, potentially decreasing by 60%, leading to an increase in internal soluble solutes. Under these conditions, apoplastic production of reactive oxygen species (ROS) promotes lipid peroxidation, increases electrolyte leakage, and increases the quantity of malondialdehyde (Møller et al., 2007). In early drought stress, root expansion was recorded. However, with the extension of drought stress, root expansion, plant growth, and plant reproduction are restrained (Wilkinson and Davies, 2010). Transpiration is an important variable in crop drought tolerance. For instance, rice and wheat drought-tolerant varieties were improved by maximizing mesophyll conductance (gm) and minimizing stomatal conductance (gs). Thus, improved transpiration efficiency is achieved. Basically, low stomatal density accompanied by a thick mesophyll and thin cell walls is considered a measurable variable to develop drought-tolerant varieties (Ouyang et al., 2017).

Abscisic acid (ABA) is also called a stress hormone and is a key hormone involved in abiotic stress resistance in plants, coordinating an array of functions (Wani and Kumar, 2015). ABA accumulation prompts stomatal closure to avoid water loss through transpiration and induces changes in gene expression (de Dorlodot et al., 2007; Finkelstein et al., 2008; Harris, 2015). Since the discovery of ABA, many experiments have been conducted to understand its synthesis and function under stress conditions. The ABA signaling pathway is composed of three main sections, pyrabactin resistance (PYR)/pyrabactin resistance-like (PYL)/regulatory component of ABA receptors (RCAR). It is also regulated by two important enzyme families, protein phosphatase 2C (PP2C, a negative regulator) and sucrose non-fermenting (SNF1)-related protein kinase 2 (SnRK2, a positive regulator). Under stress conditions, the accumulation of ABA and the formation of the PYR/PYL/RCAR-PP2C complex lead to PP2C inactivation, followed by SnRK2 activation. Activated SnRK2 facilitates the transcription of ABA-responsive genes by phosphorylating downstream substrates (Sah et al., 2016). Thus, ABA signaling is activated.

The basic leucine zipper (*bZIPs*), a transcription factor dependent on ABA, is considered to play an important role in plant response to water deficits (Xiang et al., 2008; Lu et al., 2009; Liu et al., 2014). There are 75 *bZIP* gene family members reported in the *Arabidopsis* genome (Jakoby et al., 2002). Among these genes, ABA-responsive element-binding (AREB) is an important *cis*-acting element in ABA-responsive gene expression, which increases drought stress tolerance at the transcriptional and posttranscriptional levels (Nakashima et al., 2014). A previous study shows that two AREB motifs in the promoter of the *responsive to dehydration* (*RD29B*) gene are important *cis*-acting elements that enhance ROS scavenging and osmotic adjustment (Yang and Tan, 2014). The overexpression of the *bZIP* family genes *ABF3* and *ABF4* significantly increased drought tolerance in *Arabidopsis* (Kang et al., 2002). Likewise, some *bZIP* genes have been reported to respond to drought stress in rice (Xiang et al., 2008; Lu et al., 2009; Liu et al., 2014), soybean (Yang et al., 2020), cotton (Liang et al., 2016) and other crops. In addition, *HvZIP1* is associated with the adaptability to water deficits in barley (Mezer et al., 2014).

Barley (*Hordeum vulgare* L.), the fourth most important cereal crop in the world, originating in the Middle East with a desert climate. Wild barley (*Hordeum spontaneum*) has maintained aptitudes to survive hard environmental conditions. Because wild and cultivated barley share a common genome and are cross compatible, valuable alleles are available in the wild barley to breeders for crop improvement (Dai et al., 2012; Zeng et al., 2015, 2016; Sallam et al., 2019). Several studies have been carried out in wild barley to understand its capacity to survive under abiotic stresses. As a result, a list of important enzymes affected by water deprivation has been identified, including superoxide dismutase, ascorbate peroxidase and catalase (Sallam et al., 2019). Wang et al. (2018) identified 77 different genes related to drought tolerance in wild barley. Among this pool of genes, there are genes coding for PP2C, such as MIZUKUSSEI 1 (involved in lateral root development by maintaining auxin levels), alkaline neutral invertase CINV2 (regulator of sugar-mediated root development by controlling sucrose catabolism in root cells) and *ECERIFERUM 1* (involved in epicuticular wax biosynthesis). Recently, *HvMYB1* and *HvSNAC1* have been isolated from more than 39,000 genes from barley, and their expression has been shown to confer enhanced drought tolerance to plants (Al Abdallat et al., 2014). Additionally, low stomatal density has been shown to promote better performance under water-limited conditions *via* the overexpression of *epidermal patterning factor* (*HvEPF1*) without adverse effects on normal growth (Hughes et al., 2017). Nevertheless, there are likely many of these genes that have not been fully explored.

Here, transcriptome and WGCNA analyses were performed to compare the molecular mechanisms responding to drought between wild and cultivated barley. We focused on cultivated barley Tadmor (drought-tolerance), Baudin (drought-sensitive) and wild barley EC_S1 (drought-tolerance) and identified drought-related transcription factors and hub genes. *HvbZIP21*, one of hub gene, was highly expressed in drought tolerant genotypes, therefore, we speculate that it may play a role in drought response by involving in ROS scavenging. To prove this hypothesis, the *HvbZIP21* was overexpressed in *Arabidopsis* and silenced in wild barley to verify its effect on promoting drought tolerance.

## Materials and Methods

### Plant Growth and Drought Stress Treatment

Seeds from the wild barley EC_S1 (drought-tolerant), the cultivated barley Tadmor (drought-tolerant) and Baudin (drought-sensitive) were washed with clean water for 12 h and then soaked in anhydrous ethanol for 30 seconds and 1% sodium hypochlorite for 5 min. Seeds were covered by wet filter papers and germinated in a growth chamber at 25°C and 60% relative humidity (RH). Germinated seeds were sown into plastic pots (9 cm top diameter, 5.5 cm bottom diameter, 8 cm height) filled with 120 g of 3:1 well mixed substrate and vermiculite. Seedlings were then placed into the growth chamber at 20/18°C (day/night) and under natural illumination of 12/12 h day/night cycle. During this stage, 200 ml Hoagland nutrient solution was provided every 3 days until the two-leaves stage. Before drought stress treatment, all seedlings were calibrated for water by weighing. Then, they were divided into two treatments. As a control, the plants were watered every other day. Meanwhile, in the drought-stressed treatment, the water supply was gradually eliminated so that the soil became arid and the relative air humidity was controlled at 40%. Eighteen days after drought treatment, plants were rewatered for recovery.

### Data Collection of Biomass and Stomatal Morphology

Data collection was carried out at four time points, i.e., before drought treatment (control treatment, S0), 14 days after drought treatment (S1), 16 days after drought treatment (S2), 1 day after rewatering (recovery period, R1). Plant fresh weight (FW) was determined by weighing the aboveground and underground parts of each plant separately. The turgid weight (TW) was measured by placing aboveground and underground parts into distilled water of 20°C for 8 h until saturated. Plant samples were then placed into kraft bags, and dried in an oven at 105°C for 30 min, followed by 2 weeks at 75°C to estimate the dry weight (DW). The relative water content (RWC) was calculated according to previous methods (Schonfeld et al., 1988) as following:


$$R\,W\,C\%=\frac{\left(F\,W-D\,W\right)}{\left(T\,W-D\,W\right)}\times 100$$

For stomatal morphology, the polish (acetone and resin as the main components) was spread on the lower surface of the middle part of the leaf for 2 min. The solidified nail varnish sheet was carefully separated from the leaf using tweezers and then observed and photographed under a microscope (ECLIPSE E600, Japan). The pictures were imported into the ImageJ@1.8.0 software for statistical analysis of multiple parameters analysis. Stomatal size (SS, the total area of stoma), stomatal perimeter (SP, the total length of outer border), stomatal width (SW, the top to the bottom of stoma outer border), stomatal length (SL, the left to the right of stoma outer border), stomatal aperture (SA, the pore area between the upper and lower guard cells) and aperture perimeter (AP, the total length of the internal border) were recorded.

### Antioxidant Enzyme Activity and Malondialdehyde Content

The aboveground part of the plants was sampled at the above-mentioned four time points (S0, S1, S2, and R1) and covered in tinfoil and then soaked in liquid nitrogen for antioxidant enzyme activity detection. The SOD, POD, CAT activities and MDA content were estimated according to the previously reported method with slight modification (Denaxa et al., 2020). Briefly, phosphate buffer (PBS, pH = 7.0) was used to extract the initial enzyme solution. The SOD reaction mixture consists of 1.5 ml PBS (0.05 M, pH 7.8), 0.3 ml methionine (130 mM), 0.3 ml nitroblue tetrazolium (NBT, 750 μM), 0.3 ml EDTA-Na2 (100 μM), 0.3 ml riboflavin (VB2, 20 μM) and 0.25 ml ddH2O and it was detected at 560 nm with kinetic scan for 3 min. The POD reaction mixture consists of 2.5 ml PBS (0.1 M, pH 6.0), 2.8 μl guaiacol and 19 μl H_2_O_2_ (30%), and it was detected at 470 nm with a kinetic scan for 3 min. CAT reaction mixture consists of 0.5 ml H_2_O_2_ (0.1 M) and 2 ml PBS (0.1 M, pH 7.0) and it was detected at 240 nm with a kinetic scan for 3 min. For the determination of MDA content, thiobarbituric acid was dissolved in 10% trichloroacetic acid (TCA) until a concentration of 0.6% was obtained. Then, 1 ml of initial enzyme solution was added to an aliquot of 2 ml of the mixture. MDA content was then measured at 600, 532, and 450 nm by a spectrophotometer (UV-1800, Shimadzu, Japan). To detect the presence of O_2_^–^, leaves were soaked in 2 mM nitro-blue tetrazolium (NBT), dissolved in 20 mM phosphate buffered solution (PBS, pH 6.8) for 6 h. Buffer containing 1% (w/v) of 3-diaminobenzidine (DAB, pH 3.8) was used for the detection of hydrogen peroxide (H_2_O_2_). The GSH, AsA, O_2_^–^ and H_2_O_2_ contents were measured following the methods reported previously (Yi et al., 2016).

### RNA Extraction and Sequencing

Plant samples were collected as described above using, three replicates for each barley genotype, with a total of 36 samples used for RNA sequencing. Total RNA was extracted using the MiniBEST Plant RNA Extraction Kit (Takara, Japan). The RNA Nano 6000 Assay Kit (Agilent Technologies, CA, United States) was used to detect RNA integrity. After extraction, the total RNA was enriched with oligo (DT) magnetic beads. Fragment buffer was added to break the mRNA into short fragments. The first cDNA strand was synthesized with random hexamer primers using mRNA as a template. Then, the second cDNA strand was synthesized by adding buffer, dNTPs, RNase H and DNA polymerase I. After purification by QiaQuick PCR kit and elution with EB buffer, terminal repair and sequencing connectors were performed. Then, agarose gel electrophoresis was used to select fragment sizes. Finally, after PCR amplification, the cDNA library was sequenced with Illumina HiSeq™ (Supplementary Figure 1).

### Transcriptome Analysis

The raw data were filtered with *fastp* (Q < 20 and length < 50 bp, adaptor sequences at the 3′ end) and quality control with *fastQC* to yield clean data. The genome of barley cv. Morex^1^ was used for clean read mapping by *HISAT2*. The mapping files were compressed and sorted by *Samtools*. Then, *StringTie* was used for reads assembly and abundance estimation. The fragments per kilobase of exon per million fragments mapped reads (FPKM) and false discovery rate (FDR) were used for the identification of differentially expressed genes (DEGs) with *DESeq2* (fold change ≥2 and FDR ≤0.05). Gene Ontology (GO) and Kyoto Encyclopedia of Genes and Genomes (KEGG) analyses of DEGs were performed by *clusterProfiler* with FDR ≤ 0.05 (Yu et al., 2012).

### Weighted Gene Correlation Network Analysis

All DEGs were merged into a matrix as the input data for the R (v4.0.3) package WGCNA (v1.69). All phenotypic data recorded from plants under drought stress were also imported into the R software as the targets associated with gene co-expression modules. According to the approximate scale-free topology preconditions (*R*^2^ = 0.9), the WGCNA parameters of the soft threshold power of the adjacency matrix were defined as 9. The clustering of module eigengenes was cut at a height of 0.25. The networks correlated with agronomic traits were identified with the criterion of stability correlation *P* ≤ 0.05, and the module with ≥ 0.6 correlation coefficient (Pearson’s correlation coefficient) of traits were used for further analysis. The node and edge of genes in the selected module were loaded in *Cytoscope@3.7.2* and analyzed by *cytoHubba* with Degree value, of which the notes with high connectivity within modules were considered as the hub genes.

### Real-Time Quantitative PCR

The *Primer Premier 6.0* software was used to design primers for candidate DEGs (Supplementary Table 1). Total RNA was extracted by the MiniBEST Plant RNA Extraction Kit (Takara, Japan), and its quality was assessed using a NanoDrop 2000 device (Thermo, United States) and through 1% agarose gel electrophoresis. Selected samples were reverse transcribed into cDNA by the PrimeScript™ RT reagent kit (Takara, Japan). QuantStudio 6 Flex Real-Time PCR System (Thermo, United States) was used to collect fluorescence quantities with the reaction system of 10 μL of 2 × Powerup SYBR Master Mix (Thermo, United States), 0.5 μL of forward and reverse primers, 3 μL of diluted cDNA and 6 μL DEPC-treated water. Relative quantitation was calculated according to the 2^–ΔΔ*CT*^ method.

### Phylogenetic Analysis

For phylogenetic analysis, the sequences of known *bZIP* transcription factors from *Arabidopsis*, rice, *Zea mays* and wheat were downloaded from the UniProt website.^2^ All bZIP protein sequences were aligned using ClustalW in MEGA7 software, and then an unrooted phylogenetic tree was constructed by the neighbor-joining algorithm with a bootstrap value of 1,500 and the selection parameter *p*-distance.

### Overexpression of HvbZIP21 in Arabidopsis

Sequences of the *HvbZIP21* genes from a reference genome (assembly of EC_S1, unpublished) were used for primer design. The coding regions were amplified from EC_S1 cDNA, ligated into a pMD18-T vector and transferred into *Escherichia coli* competent cells for sequencing. The coding regions were then transferred into the pNC-Cam1304-MCS35S binary vector (Supplementary Figure 2) by homologous recombination. The recombined vector was transferred into *Agrobacterium tumefaciens* GV1301 competent cells. The flower buds of *Arabidopsis* were infected by *Agrobacterium* liquid buffer. Hygromycin B, GFP excitation light source and molecular marker for PCR (Supplementary Table 1) were used to identify the plants where the recombinant vector was integrated. RT-qPCR was used to confirm the presence and expression levels of candidate genes. Finally, homozygous transgenic seeds were sown into pots for 2 weeks. Water was withheld from plants in the drought stress treatment, while 100 ml of water was provided to plants in the control treatment. After 16 days, the phenotypic and physiological parameters were collected.

### VIGS and Drought Tolerance Assessment

Barley stripe mosaic virus (BSMV) was used for gene silencing (Supplementary Figure 3). A fragment of 242 bp of *HvbZIP21* was amplified by PrimeSTAR^®^ Max DNA Polymerase (Takara, Japan) and then ligated into the pMD18-T vector for sequencing. To avoid off-target effects, the sequence within 3′UTR region was cloned as target fragment. Then, the target sequence was further run by “Blastex” in reference genome (wild barley EC_S1), which suggested that no homologous fragment was detected at E value < 0.05 or identity > 50%. Afterward, RNAγ cDNA was treated with *NheI* endonuclease, and the target sequence was amplified by primers harboring a homologous fragment of *NheI* sides 20 bp. Finally, the target sequence was inserted into RNAγ cDNA by In-Fusion Snap Assembly Master Mix (Takara, Japan). The combined RNAγ:*HvbZIP21* with reverse insertion was used to silence *HvbZIP21*. During these steps, *HvPDS* was also cloned and used as a positive control to test the silencing effect.

The cDNA of RNAα, RNAγ, RNAγ:*HvbZIP21* and RNAγ:*HvPDS* was linearized with *MluI*, and RNAβ was linearized with *SpeI*. *In vitro* RNA synthesis was conducted with a T7 RiboMAX™ Express Large Scale RNA Production System kit and Ribo m7G Cap kit (Promega, United States) based on the manufacturer’s instructions. The mixture solution, containing RNAα, RNAβ and RNAγ/RNAγ:*HvbZIP21*/RNAγ:*HvPDS* (1:1:1 in volume) was three times diluted with RNase-free water, and then equal 2 × GKP buffer was added. A total of 8 μl of the final mixture was inoculated into EC_S1 seedling leaves at the two-leaf stage according to the previously reported approach (He et al., 2015). Three inoculation treatments were conducted, i.e., RNAα + RNAβ + RNAγ; RNAα + RNAβ + RNAγ:*HvbZIP21*; and RNAα + RNAβ + RNAγ:*HvPDS*. The experiment was performed in three replicates using 10 plants (five plants from each of drought stress and control treatments) for each replicate. The drought treatment was the same as the methods described before.

### Statistical Analysis

Analysis of variance (ANOVA) was performed using IBM^®^ SPSS^®^ Statistics 20. Sample groups with significantly different averages were analyzed further using Fisher’s least significant difference (LSD) test at a 5% probability (*P*-value ≤ 0.05) level (IBM^®^ SPSS^®^ Statistics 20).

## Results

### The Growth of Wild Barley and Cultivated Barley Under Drought Stress

**FIGURE 1:**
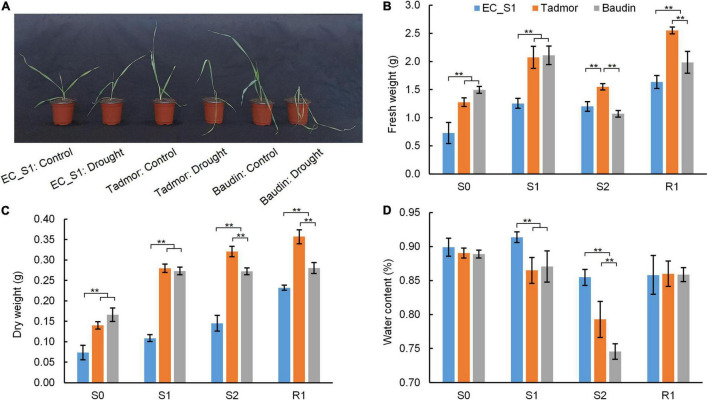
The morphology and biomass of EC_S1, Tadmor and Baudin after drought treatment for 16 d. **(A)** Photo of plants under control and drought treatment conditions; **(B)** fresh weight; **(C)** dry weight; **(D)** water content. S0: drought for 0 day; S1: drought for 14 days; S2: drought for 16 d; R1: drought for 18 days and rewatered for 1 day. * and ** indicate significance levels at *P* ≤ 0.05 and 0.01, respectively (*n* = 3 independent replicates).

Water deficiency will reduce the metabolic reaction rate of crops and inhibit their growth. Compared to the well-watered treatment, leaves of the cultivated barley Baudin appeared wilted and yellow under drought conditions, and the leaves of cultivated barley Tadmor were slightly curled, whereas the leaves of the wild barley EC_S1 showed almost no significant change (Figure 1A). The fresh weights of EC_S1, Tadmor and Baudin increased by 72.5, 62.6, and 41.1%, respectively, after 14 days under drought stressed conditions. However, prolonging the drought stress conditions to 16 days has decreased the fresh weights of EC_S1, Tadmor and Baudin by 4.2, 25.3, and 49.2%, respectively. During the recovery stage, the fresh weights of EC_S1, Tadmor and Baudin were increased by 36.2, 64.7, and 85.2%, respectively (Figure 1B). The dry weights were significantly increased in EC_S1 (47.9, 33.8, and 60.1%) and Tadmor (100.1, 14.7, and 11.4%) under drought stress for 14 and 16 days and 1 day after recovered, respectively. However, the dry weight of Baudin was significantly increased by 64.4%, under drought conditions for 14 d, while there were no obvious changes under 16 days of drought stress and 1 days after recovery (Figure 1C). Moreover, water contents of Tadmor and Baudin were slightly decreased by 2.8 and 2.1%, respectively, 14 days after drought stress and significantly decreased by 8.4 and 14.4% after 16 days of drought stress, however, it was increased by 8.4 and 15.2% after 1 days of recovery. The water content of EC_S1 remained at 79.3–86.5% during drought stress (Figure 1D). These results indicated that under drought stress, the reduction in water content was the most serious in the Baudin genotype, and therefore, plant growth was significantly inhibited, while EC_S1 and Tadmor genotypes were more tolerant to drought stress.

### Genotypic Differences in Stomatal Morphology Under Drought Treatment

**FIGURE 2:**
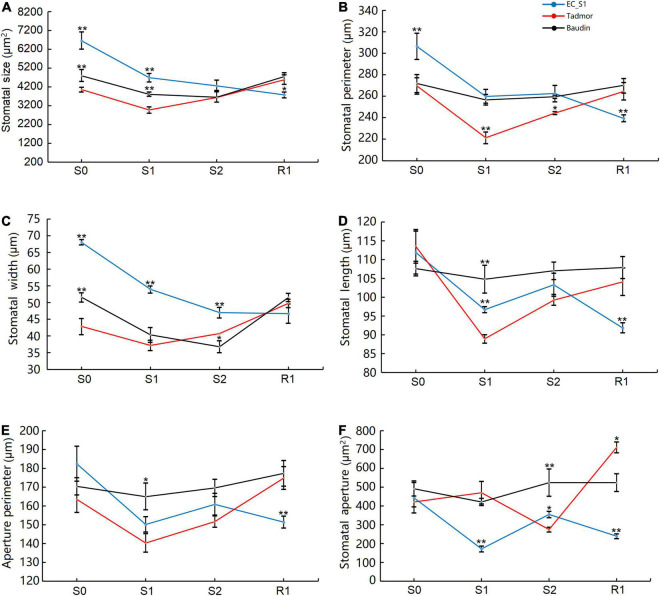
Stomatal morphology of EC_S1, Tadmor and Baudin after drought treatment. **(A)** Stomatal size; **(B)** stomatal perimeter; **(C)** stomatal width; **(D)** stomatal length; **(E)** aperture perimeter; **(F)** stomatal aperture. * and ** indicate significance levels at *P* ≤ 0.05 and 0.01, respectively (*n* = 3 independent replicates).

Stomata regulate photosynthesis by changing the concentration of intercellular carbon dioxide, and change the plant water content by affecting the transpiration rate. This behavior is one of the most important response strategies of plants in response to drought stress. The wild barley EC_S1 showed larger SS, SP and SW compared to the cultivated barley Baudin and Tadmor. Moreover, SS, SP and SW in the EC_S1 were significantly decreased by 29.6, 17.7, and 20.7%, respectively, after 14 days of drought treatment, then reached the lowest values after recovery. In Tadmor, SS, SP and SW significantly decreased by 26.8, 18.0, and 13.3%, respectively, after 14 days of drought treatment, and then returned to normal their levels after recovery. However, SS and SP did not differ significantly in the Baudin genotype under the drought stress and recovery stage, whereas SW was significantly decreased by 21.8 and 8.9% after 14 and 16 days of drought stress, respectively, and then was increased to its normal level (Figures 2A–C). SL and AP exhibited no significant changes in Baudin but first decreased and then increased in Tadmor and fluctuated in EC_S1 (Figures 2D,E). The SA was significantly decreased in the Tadmor genotype after 16 days of drought stress and increased after 1 day of recovery. However, in EC_S1 genotype, SA was significantly decreased by 61.5% after 14 days and increased at 16 days of drought treatment. There were no obvious changes in SA in the Baudin genotype in response to drought stress (Figure 2F). These results suggest that Baudin had insensitive stomatal regulation, which made Baudin a drought-sensitive genotype. Additionally, differences between the wild barley EC_S1 and the cultivated barley Tadmor in their response to drought stress with respect to measured traits were also observed. In details, Tadmor has smaller initial stomatal morphological parameters than the other two barley genotypes, while EC_S1 genotype has a more flexible stomatal regulation strategy, which can rapidly regulate stomatal aperture to a tiny size under drought stress, although it has larger initial stomatal morphological parameters.

### The Activity of Antioxidant Enzymes and MDA Content Under Drought Stress

**FIGURE 3:**
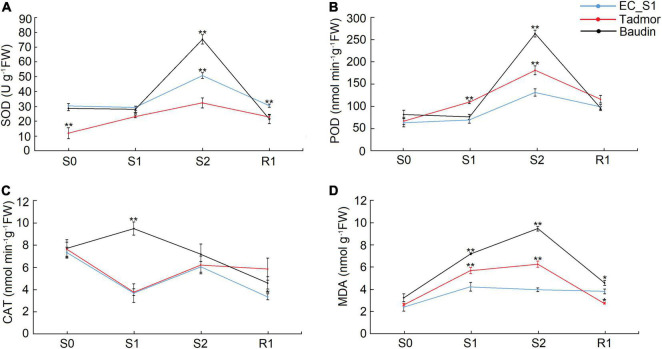
The activity of enzymes related to ROS scavenging and MDA content of EC_S1, Tadmor and Baudin after drought treatment. **(A)** SOD activity; **(B)** POD activity; **(C)** CAT activity; **(D)** MDA content. * and ** indicate significance levels at *P* ≤ 0.05 and 0.01, respectively (*n* = 3 independent replicates).

Abiotic stress usually causes an increase in reactive oxygen species (ROS) in plants. In response, plants increase the activity of antioxidant enzymes to eliminate reactive oxygen species and avoid cellular damage. The SOD and POD activities of all three barley genotypes showed increasing levels, peaked at 16 days after drought stress and decreased during the recovery stage. In addition, Baudin had the highest SOD and POD activities among the three barley genotypes, while the lowest SOD activity was found in the Tadmor genotype and the lowest POD activity was detected in EC_S1 under drought stress (Figures 3A,B). The CAT activity of Baudin peaked at 14 days of drought treatment, and it decreased after 14 days but increased after 16 days of drought treatment in Tadmor and EC_S1 (Figure 3C). The MDA content was significantly increased under drought treatment for 14 and 16 days, and then decreased during the recovery stage in Baudin and Tadmor. However, the MDA content of EC_S1 showed did not change significantly under either the drought stress and recovery stages (Figure 3D). These results suggest that more ROS are produced in Baudin plants and induce higher activity of antioxidant enzymes, however, it also had higher MDA accumulation than the other two genotypes. Moreover, EC_S1 exhibited the lowest MDA accumulation, which may be an important reason for its strong drought tolerance.

### Identification of DEGs in Barley Genotypes Under Drought Stress

**FIGURE 4:**
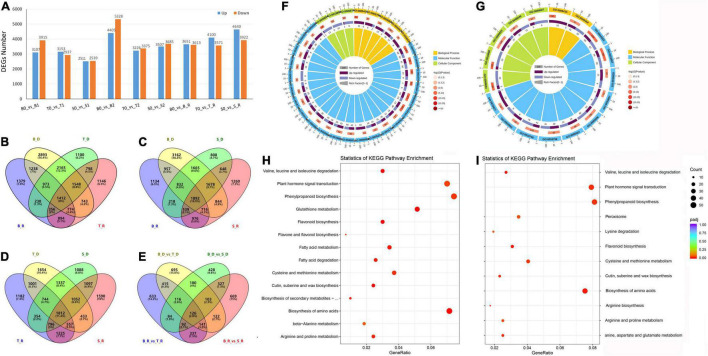
Identification and functional annotation of DEGs in EC_S1, Tadmor and Baudin under drought treatment. **(A)** DEGs number; B0: 0 day of drought treatment in Baudin; B1: 14 days of drought treatment in Baudin; B2: 16 days of drought treatment in Baudin; B_R: 18 days of drought treatment and 1 days of rewatering in Baudin; S: EC_S1; T: Tadmor; Venn of DEGs in drought and rewater stage of Baudin and Tadmor **(B)**; Baudin and EC_S1 **(C)**; Tadmor and EC_S1 **(D)** Baudin_vs_Tadmor and Baudin_vs EC_S1 **(E)**; B_D and B_R: DEGs of Baudin in drought and rewater treatment; S_D and S_R: DEGs of EC_S1 in drought and rewater treatment; T_D and T_R: DEGs of Tadmor in drought and rewater treatment; B_D_vs_T_D: DEGs in Tadmor compared to Baudin under drought treatment; B_D_vs_S_D: DEGs in EC_S1 compared to Baudin under drought treatment; **(F)** GO annotation of DEGs in Tadmor; **(G)** GO annotation of DEGs in EC_S1; **(H)** KEGG enrichment of DEG in Tadmor; **(I)** KEGG enrichment of DEG in EC_S1.

When plants sense drought signals, the expression levels of specific genes are induced, resulting in a series of physiological and metabolic reactions. In this study, approximately 283.09 GB of clean data were used for alignment and quantification analysis (Supplementary Table 2). A total of 7,022 (3,107 up-regulated and 3,915 down-regulated), 6,090 (3,153 up and 2,937 down-regulated) and 5,050 (2,511 up and 2,539 down-regulated) DEGs were detected in Baudin, Tadmor and EC_S1, respectively, after 14 days of drought treatment. In addition, 9,733 (4,405 up and 5,328 down-regulated), 6,601 (3,226 up and 3,375 down-regulated) and 7,192 (3,507 up and 3,685 down-regulated) DEGs were detected in Baudin, Tadmor and EC_S1, respectively, after 16 days of drought stress. Obviously, the expression levels of more genes were changed in the drought-sensitive barley genotype Baudin, meaning that the drought-tolerant barley genotypes have more stable gene regulation. In addition, the wild barley EC_S1 has delayed gene regulation compared to cultivated barley Tadmor, although both genotypes were high drought-tolerant. However, during the recovery stage, there were 7,264 (3,651 up and 3,613 down-regulated), 7,671 (4,100 up and 3,571 down -regulated) and 8,562 (4,640 up and 3,922 down -regulated) DEGs in Baudin, Tadmor and EC_S1, respectively (Figure 4A). These results suggest that compared to drought-sensitive genotype, drought-tolerant barley genotypes have more active gene regulation, and that the wild barley genotype was more active compared to cultivated barley genotypes under the recovery stage.

To reveal the difference in drought tolerance mechanisms between wild and cultivated barley, DEGs in the drought-tolerant genotype EC_S1 and Tadmor were compared to those of the drought-sensitive genotype Baudin. Under drought stress and the recovery stage, 1,898 and 1,944 DEGs, respectively, were identified in Tadmor (named T-Drought DEGs), including 798 common genes (Figure 4B). In EC_S1, 1,456 and 1,917 DEGs were detected (named W-Drought DEGs), including 648 common genes (Figure 4C). These results suggest that fewer specific DEGs were detected in EC_S1 than in Tadmor under drought treatment. Moreover, 4,945 and 4,390 DEGs were common genes under drought stress and during the recovery stage. In addition, 2,655 and 2,185 DEGs were identified in Tadmor and EC_S1 under drought stress, respectively, and 2,183 and 2,687 DEGs were identified during the recovery stage (Figure 4D). Among the T-Drought DEGs and W-Drought DEGs, 1,110 and 1,048 DEGs were identified in Tadmor under the drought stress and recovery stages, respectively, from which 415 DEGs were common genes. In contrast, 755 and 996 DEGs were identified in EC_S1, including 327 common genes (Figure 4E). In general, compared to cultivated barley, the number of specific DEGs in wild barley under drought stress was lower but was equivalent to that in cultivated barley under the recovery period, meaning that the regulation of wild barley in response to drought may be stable and efficient.

DEGs in the wild barley EC_S1 and cultivated barley Tadmor were annotated by Gene Ontology (GO) annotation. In total, 30 GO terms, including 3 terms in cellular components, 5 terms in biological processes and 22 terms in molecular function, were significantly enriched in Tadmor. In contrast, only 16 GO terms in EC_S1 were enriched, including 3 terms in cellular components, 2 terms in biological processes and 11 terms in molecular function. The DEGs of Tadmor were significantly enriched in the GO terms “hydrolase activity (GO: GO:0004553),” “response to water (GO:0009415)” and “transmembrane transporter activity (GO:0022857)” and EC_S1 in “hydrolase activity (GO:0004553),” “photosystem II oxygen evolving complex (GO:0009654)” and “transmembrane transporter activity (GO:0022857).” Compared to Tadmor, EC_S1 had a significant GO term of “cell redox homeostasis (GO:0045454)” in biological process, while it lacked biological processes related to cell wall and membrane degradation (GO:0006869; GO:0006629; GO:0006032; GO:0016998). Furthermore, terms related to ATP metabolism (GO:0042626; GO:0016887) and transport activity (GO:0022857; GO:0008483) were only significantly enriched in Tadmor, which indicated that the metabolic process of Tadmor was more active than that of EC_S1 under drought stress (Figures 4F,G and Supplementary Figure 4). KEGG annotation suggested that “plant hormone signal transduction (map04075)” was identified in all three barley genotypes, while “phenylpropanoid biosynthesis (map00940)” was only significantly enriched in the drought-tolerant genotypes EC_S1 and Tadmor (Figures 4H,I and Supplementary Figure 5). In addition, “peroxisome (map04146)” was only significantly enriched in EC_S1, but “glutathione metabolism (map00480)” and “flavone and flavonol biosynthesis (map00944)” were specifically enriched in Tadmor, suggesting that genes related to ROS metabolism respond differently among the three barley genotypes and may be the key factors affecting drought tolerance.

Transcription factors (TFs) are involved in the plant response to drought stress by regulating the expression level of downstream genes. Here, we identified 5 *AP2*, 1 *bZIP*, 20 *MYB*, 2 *NAC*, 3 *DREB* and 4 *WRKY* TFs (Supplementary Figures 6A–E) that were significantly induced by drought stress in the wild barley EC_S1. Moreover, the genes in known pathways were identified (Supplementary Figure 7). The genes related to stomatal closure, such as PYR/PYL and SnRK2 family genes, had obviously higher expression levels in EC_S1 and Tadmor, suggesting that drought-tolerant varieties have stronger stomatal regulation ability.

### Mining Hub Genes Involved in the Drought Stress Response

**FIGURE 5:**
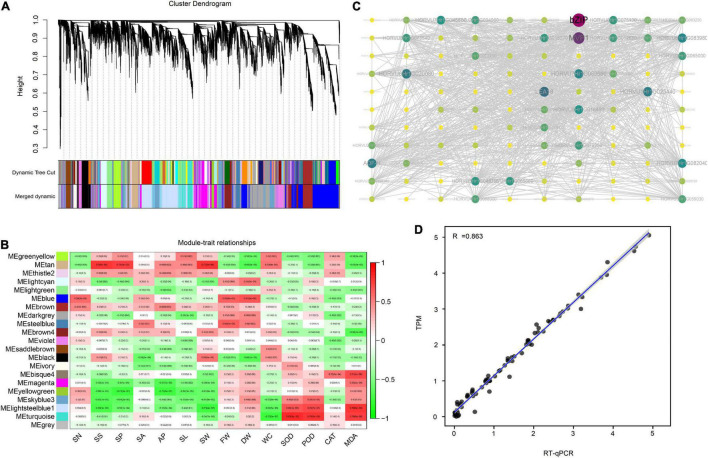
Results of Weight Gene Coexpression Network Analysis (WGCNA). **(A)** The clustering dendrogram of genes with dissimilarity based on topological overlap computed by WGCNA. The original modules and merged modules are colored below the clustering dendrogram; **(B)** the relationship between modules and traits; **(C)** the network within the Tan module. The degree value was mapped as node color and size. More modules are shown in Supplementary Figure 8. **(D)** The relationship between the transcriptome data and RT-qPCR. TPM: Transcripts per million, used to represent the expression level.

To mine the hub genes in response to drought stress, weighted gene co-expression network analysis (WGCNA) was performed to link gene expression levels with plant morphological and physiological data. The FPKM matrix from the transcriptome was input as a genotype matrix for WGCNA, and the biomass, stomatal morphological parameter, antioxidant enzyme activity and malondialdehyde content data were input as a phenotype matrix. In total, 21 modules were identified by constructing a topological overlap mapping metric (TOM) plot to calculate the similarity matrix of gene expression between two nodes. Among them, the largest module was Blue, with 3,100 DEGs, and the smallest module was Thistle 2, with 88 DEGs (Figure 5A). The drought-response modules were identified with correlation values between module and phenotype of > 0.6 and *P* < 0.05. Module Tan was considered to play a role in SS, SP and SW regulation, module Blue in FW and DW, module Steelblue in FW, module Bisque4 in MDA, and modules Lightsteelblue1 and Turquoise in SOD, POD and MDA (Figure 5B). Then, the regulatory relationship within each module was used to calculate the Degree value and construct a network. In these six modules, the genes with the top 5 Degree values were identified as hub genes in each module (Supplementary Table 3).

The hub gene in module tan includes four light-harvesting complex binding genes (*LHCB3*, *LHCB1.1*, *LHCB1.2* and *LHCB1.3*) and a *photosystem I iron-sulfur center* (*psaC*) gene, indicating that genes are involved in the photosynthesis response by regulating stomatal size, stomatal perimeter and stomatal width under drought stress. Genes in Module Blue are involved in RNA transcription (*TAF RNA Polymerase I subunit A*), protein synthesis and translocation (*protein translocase subunit*, *secA*), while genes in module steelblue are involved in ROS metabolism (*GH17B*, *POD1a*, *POD1b* and *GH17C*), suggesting that ROS, RNA and protein metabolism affect the growth of plants under drought stress. Interestingly, the hub genes of the three ROS-related modules revealed diverse functions, such as *bZIP transcription factor* (*bZIP21*), *GDSL-like* lipase/acylhydrolase (*AP22.35*), *zinc-ribbon* (*TRIM13*), and *late embryogenesis abundant* (*LEA*) *group 1* (*LEA18*), suggesting that ROS regulation is complex and is influenced by multiple pathways (Figure 5C, Supplementary Figure 8). The RT–qPCR results showed a correlation coefficient (R^2^) of 0.863 with the transcriptomic data TPM (Figure 5D), proving the reliability of the transcriptome analysis.

### HvbZIP21 Promotes Drought Tolerance in Transgenic Arabidopsis

**FIGURE 6:**
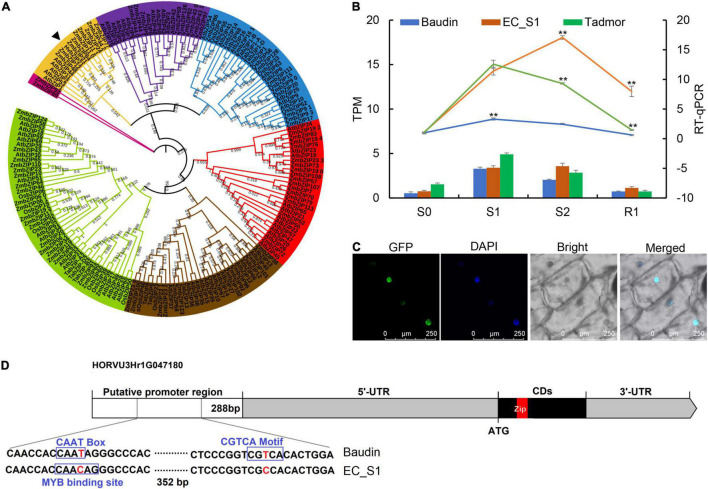
The evolution, expression pattern, subcellular localization and gene structure of *HvbZIP21*. **(A)** Phylogenetic tree constructed by the neighbor-joining algorithm with a bootstrap value of 1,500; **(B)** the expression level of *HvbZIP21* from transcriptome data (TPM) and RT-qPCR; * and ** indicate significance levels at *P* ≤ 0.05 and 0.01, respectively (*n* = 3 independent replicates). **(C)** the subcellular localization of *HvbZIP21*; **(D)** the structure of *HvbZIP21* in EC_S1.

**FIGURE 7:**
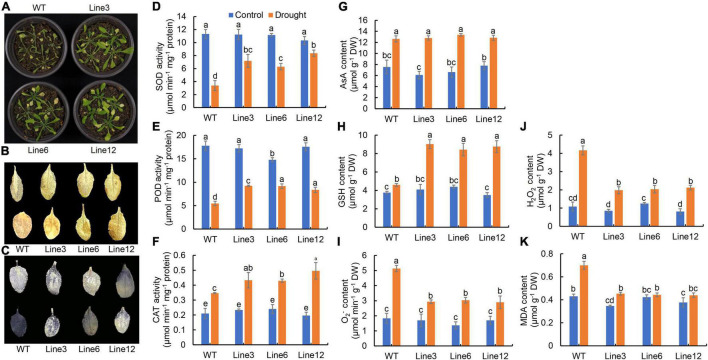
Overexpression of *HvbZIP21* in *Arabidopsis*. **(A)** Photo of WT and transgenic plants; **(B)** DAB staining of leaves of WT and transgenic plants; **(C)** NBT staining of leaves of WT and transgenic plants; **(D)** SOD activity; **(E)** POD activity; **(F)** CAT activity; **(G)** AsA content; **(H)** GSH content; **(I)** O_2_^–^ content; **(J)** H_2_O_2_ content; **(K)** MDA content. Different lowercase letters indicate significance levels at *P* ≤ 0.05 (*n* = 3 independent replicates).

A total of 30 hub genes were identified by WGCNA, including a *bZIP* transcription factor gene. The *bZIP* TF was close to *ZmbZIP21* from a phylogenetic perspective, so it was named *HvbZIP21* (Figure 6A). The expression patterns showed that *HvbZIP21* had a higher expression level in EC_S1 than in Tadmor and Baudin based on both transcriptome and RT-qPCR (Figure 6B). The full-length coding region of *HvbZIP21* was cloned from the wild barley EC_S1 (Supplementary Figure 9). The protein sequence of *HvbZIP21* was combined with GFP and subcellularly detected to the nucleus (Figure 6C). Sequence analyses found a SNP between EC_S1 (cytosine, C) and Baudin (Thymine, T). The *cis*-acting element analysis of the promoter region showed that the SNP results in a “CAAT Box” in Baudin and a “MYB binding site” in EC_S1, which may be the reason for the different expression among the three barley genotypes (Figure 6D). To reveal the function of *HvbZIP21* during drought stress, we overexpressed this gene in *Arabidopsis*. After hygromycin screening, GFP excitation light source screening and PCR identification, T3 (third-generation) plants overexpress *HvbZIP21* under the control of the cauliflower mosaic virus (CaMV) 35S promoter (35S::*HvbZIP21*; line 3, 6 and 12 were generated and subjected to drought stress for 16 days (Supplementary Figure 10). The wild-type (WT) plants were wilting and yellowing, while all three transgenic plants showed slight curl of leaf edges, suggesting that the overexpression of *HvbZIP21* improved drought tolerance in *Arabidopsis* (Figure 7A). DAB and NBT staining showed that leaves of WT plants accumulated more H_2_O_2_ and O_2_^–^ than transgenic plants (Figures 7B,C). SOD and POD activities were significantly decreased by 70.00 and 69.29% in WT plants, respectively, but 32.84 and 45.62% in transgenic plants (average data of three transgenic lines), indicating that SOD and POD activities were 2.14 and 1.63 times higher in the 35S::*HvbZIP21* transgenic plants compared to WT plants under drought treatment (Figures 7D,E). The CAT activity and AsA content were significantly increased by 65.08 and 66.96%, respectively, in the WT plants, whereas these two parameters were significantly increased by 105.81 and 91.96%, respectively, in the 35S::*HvbZIP21* transgenic plants under drought stress (Figures 7F,G). Though it was significantly increased in all plants, the GSH content in 35S::*HvbZIP21* transgenic plants was found to be 2.19 times that of the WT plants under drought treatment (Figure 7H). Consistent with the results of histochemical staining, the H_2_O_2_ and O_2_^–^ contents were significantly increased by 284.62 and 180.00% in the WT plants but by 119.66 and 88.36% in the 35S::*HvbZIP21* transgenic plants after drought treatment (Figures 7I,J), resulting in an MDA content of 1.67 times higher in the WT plants than in transgenic plants (Figure 7K). Together, the data revealed that the overexpression of *HvZIP21* enhanced the drought tolerance in *Arabidopsis* by improving the activity of oxidative protective enzymes and glutathione content.

### Silencing of the HvbZIP21 Suppresses Drought Tolerance in Wild Barley

**FIGURE 8:**
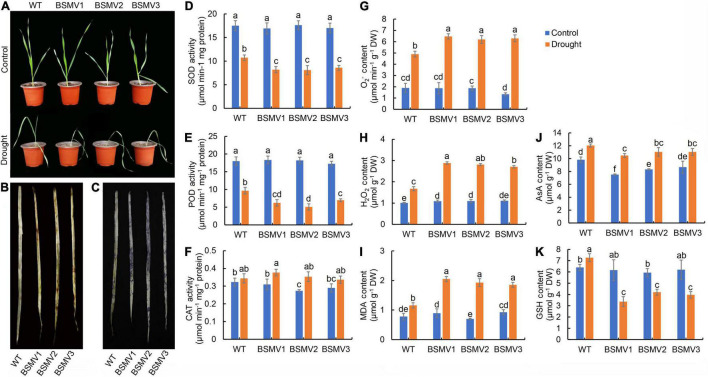
Silencing of *HvbZIP21* in EC_S1 by VIGS-BSMV. **(A)** Photo of WT and BSMV:*HvbZIP21*-inoculated plants; **(B)** DAB staining of leaves of WT and BSMV:HvbZIP21-inoculated plants; **(C)** NBT staining of leaves of WT and BSMV:HvbZIP21-inoculated plants; **(D)** SOD activity; **(E)** POD activity; **(F)** CAT activity; **(G)** AsA content; **(H)** GSH content; **(I)** O_2_^–^ content; **(J)** H_2_O_2_ content; **(K)** MDA content. Different lowercase letters indicate significance levels at *P* ≤ 0.05 (*n* = 3 independent replicates).

To verify the function of *HvbZIP21* during drought stress in barley, the gene was silenced in the drought-tolerant barley genotype EC_S1. As a control, the expression level of PDS in BSMV:*PDS*-inoculated plants was 29.1% of that in BSMV:γ-inoculated plants, causing leaf albinism in the former (Supplementary Figure 11). RT-qPCR results showed that the expression level of *HvbZIP21* was knocked down by 71 and 82% in the BSMV:*HvbZIP21*-inoculated plants compared to the BSMV:γ-inoculated plants under the well-watered and drought-stressed conditions, respectively (Supplementary Figure 12). After 16 days of drought treatment, the BSMV:γ-inoculated plants exhibited leaves drooping from the middle, but the BSMV:*HvbZIP21-*inoculated plants exhibited severe wilting and curling, and mature leaves were drooped from the base (Figure 8A). Obviously, *HvbZIP21*-silenced plants had a more drought-sensitive phenotype compared to the *HvbZIP21* non-silenced plants, suggesting that *HvbZIP21* plays a pivotal role in regulating drought tolerance in barley. DAB and NBT staining showed that the BSMV:*HvbZIP21-*inoculated plants accumulated more H_2_O_2_ and O_2_^–^ than BSMV:γ-inoculated plants (Figures 8B,C). After drought treatment, the SOD and POD activities were significantly decreased by 38.78 and 46.30%, respectively, in the BSMV:γ-inoculated plants, whereas in the BSMV:*HvbZIP21*-inoculated plants, the SOD and POD activities were decreased by 51.72 and 65.94%, respectively (Figures 8D,E). The CAT activity and AsA content were significantly increased in both BSMV:γ- and BSMV:*HvbZIP21*-inoculated plants under drought stress (Figures 8F,G). Interestingly, the GSH content was significantly increased by 13.54% in BSMV:γ-inoculated plants, but it was significantly decreased by 36.88% in the BSMV:*HvbZIP21*-inoculated plants in response to drought stress (Figure 8H). As a result, H_2_O_2_ and O_2_^–^ contents in the BSMV:*HvbZIP21*-inoculated plants were 1.67 and 1.29 times those of BSMV:γ-inoculated plants, respectively (*P* < 0.05, Figures 8I,J). Finally, the MDA content of BSMV:*HvbZIP21-inoculated plants* was 1.68 times higher than that of the BSMV:γ-inoculated plants (Figure 8K). These results show that silencing the *HvbZIP21* reduces the activity of oxidative protective enzymes, and the glutathione content in BSMV:*HvbZIP21-* and BSMV:γ-inoculated plants showed a completely opposite trend under drought stress. Finally, cell membrane peroxidation caused by the rise in ROS content inhibited growth and physiological metabolic activity in the BSMV:*HvbZIP21*-inoculated plants. The above results support the idea that *HvbZIP21* is involved in drought tolerance in barley by regulating the level of ROS.

## Discussion

Plant responses to drought stress can be divided into three strategies: escape, avoidance and tolerance (Gupta et al., 2020). Previous studies have shown that wild barley from the desert areas has stronger drought tolerance (reduced water loss) than wild barley from the Mediterranean grassland areas (Shahmoradi and Mozafari, 2015). In this study, we found that the water content was low in the drought-sensitive Baudin barley and high in the drought-tolerant wild barley EC_S1. This finding suggests that drought avoidance (defined as endurance with increased internal water content and prevention of tissue damage) was an effective strategy for wild barley to cope with drought conditions. Stomatal size, which is negatively correlated with plant drought tolerance, directly affects the plant’s water content by regulating transpiration (Zhou, 2008; Chen et al., 2018). Interestingly, the initial stomatal size (SS), stomatal perimeter (SP) and stomatal width (SW) of the wild barley were significantly higher than those of cultivated barley (Figures 2A–C), but the stomatal length (SL), aperture perimeter (AP) and stomatal aperture (SA) were not significantly different (Figures 2D–F). This can be explained by the morphology of the guard cells, which are larger in size and width in the wild barley EC_S1compared to cultivated barley. Under drought conditions, the stomatal regulation in the wild barley EC_S1 showed two features: (1) a faster response rate and greater amplitude change than cultivated barley; and (2) changes in volatility to balance drought tolerance and growth. These results are in contrast with a previous study, where stomatal aperture width, stomatal density, and stomatal pore area were significantly lower in the drought-sensitive wild barley varieties (Tibetan) (Chen et al., 2018). We speculate that a potential reason for these contrasting results may involve data being sourced from a single time point, whereas stomatal regulation changes dynamically. Importantly, other studies found that drought-tolerant barley varieties had larger guard cell volumes, which is consistent with our results. The observed low values of most stomatal parameters in the drought-tolerant cultivated barley Tadmor under drought stress might be associated with effectively reduction in water loss. Besides, the non-significant changes in stomatal aperture of the drought-sensitive cultivated barley Baudin under drought stress might be the reason for its weak drought tolerance.

Transcriptome analysis has been widely used to mine drought tolerance genes in barley (He et al., 2015; Zeng et al., 2016; Harb et al., 2020), but only a few studies have focused on wild barley from the Middle East. In our study, the number of differentially expressed genes in drought-tolerant barley (6,090 in Tadmor and 5,050 in EC_S1) was significantly lower than that in drought-sensitive barley (7,022 in Baudin) (Figure 4A). These results support the conclusion of previous studies that, compared to drought-sensitive barley genotypes, drought-tolerant barley genotypes have more stable gene expression changes (Janiak et al., 2017). GO annotation analyses also revealed differences between wild and cultivated barley. The plants of the tadmor variety showed strong energy metabolism (ATP metabolism GO:0042626; GO:0016887 and transport activity GO:0022857; GO:0008483), while plants of EC_S1 showed stable redox metabolism (cell redox homeostasis GO:0045454) (Figures 4F,G). KEGG annotation supported similar results, showing that “peroxisome (map04146)” was only significantly enriched in EC_S1 (Figures 4H,J). Interestingly, oxidative protective enzymes, such as SOD, POD and CAT, were higher in the Baudin variety than Tadmor and EC_S1. Likewise, the Baudin variety also exhibited the highest MDA content among the three barley genotypes (Figures 3A–D). The SOD, POD and CAT activities in EC_S1 genotype were significantly lower than those in Baudin, which may be caused by low ROS accumulation in EC_S1. Among the three varieties, the lowest MDA content was observed in EC_S1, suggesting that, compared to Baudin and Tadmor, the cell membrane of EC_S1 was less damaged by ROS accumulation under drought stress.

Through the WGCNA R package (Zaidi et al., 2020), we identified 30 hub genes, including nine known drought-related genes. For example, the *LHCB6* gene in *Arabidopsis* has been reported to play a role in stomatal regulation by responding to ABA signaling in guard cells, and ROS may be involved in this process (Xu et al., 2012). Here, we have identified 4 *LHCB* family genes (*HORVU2Hr1G040780*, *HORVU7Hr1G058120*, *HORVU5Hr1G066280* and *HORVU1Hr1G088920*) related to stomatal parameters, suggesting that the *LHCB* gene family may also play a role in regulating drought response in barley (Supplementary Figure 6E). Moreover, two peroxidase family genes (*HORVU2Hr1G018480* and *HORVU2Hr1G044340*) were identified as hub genes (Supplementary Figure 6D). Previous studies have shown that the overexpression of extracellular peroxidase *CaPO2* in *Arabidopsis* enhanced tolerance to drought stress (Choi and Hwang, 2012). The hub genes of three modules related to oxidative protective enzyme activity contained some known drought-related genes, such as *LEA18* (*HORVU6Hr1G054890*), *ABCB6* (*HORVU4Hr1G000620*) and *bZIP* transcription factor (*HORVU3Hr1G047180*) (Supplementary Figure 6). The overexpression of *group 2 of late embryogenesis assistant* (*LEA*) in tomato significantly improved its drought tolerance by distributing *Na*^+^ in adult and young leaves (Munoz-Mayor et al., 2012). In addition, the *group 4 late embryogenesis abundant protein* (*LEA*) was also reported to play a role in plant tolerance to water deficit in *Arabidopsis* (Olveracarrillo et al., 2011). In a study by Kuromori et al. (2010), the *AtABCG22* mutant showed increased water loss, whereas the *AtABCG25* gene was found to be involved in stomatal regulation. In addition, *ABCG40* was identified as a plasma membrane ABA uptake transporter involved in the drought regulation (Kang et al., 2010). The identification of these drought tolerance genes not only supports the reliability of transcriptome and WGCNA analyses in candidate gene mining but also provides the implementation of these techniques as powerful tools for the identification of new drought tolerance candidate genes. In our study, we identified another 21 hub genes, but their role in the drought tolerance response is still unknown.

Transcription factors (TFs), such as *AP2*, *ERF*, *NAC*, *MYB*, *WRKY*, *DREB*, and *bZIP*, have been shown to play roles in the drought response regulation (Gujjar et al., 2014; Gupta et al., 2020; Wang et al., 2022). Here, we identified 35 TFs in total that were significantly increased in EC_S1 under drought treatment (Supplementary Figure 6). Moreover, one *bZIP* transcription factor was identified as a hub gene of the turquoise module, for which the correlation coefficients with SOD, POD and MDA were 0.83 (*P* = 3e-10), 0.79 (*P* = 9e-9) and 0.76 (*P* = 8e-8), respectively (Figure 5B). Evolutionary analyses found that this gene has the closest phylogenetic relationship to the *ZmbZIP21* gene, so it was named *HvbZIP21*. Interestingly, only *bZIP* TFs from *Arabidopsis* and *Zea mays*, but not from wheat and rice, were detected in the same cluster of *HvbZIP21* (Figure 6A), suggesting that it is a novel *bZIP* TF in barley. At present, the role of *bZIP* family genes in the drought response has been widely reported, for example, *OsbZIP23*, *OsbZIP71* and *OsbZIP72* in rice (Xiang et al., 2008; Lu et al., 2009; Liu et al., 2014) and *AREB1*, *AtTGA4*, and *AtbZIP62* in *Arabidopsis* (Furihata et al., 2000; Zhong et al., 2015; Kabange et al., 2020). However, in barley, the functions of the *bZIP* gene family under drought stress needs to be further investigated. Previous studies reported that *HvABI5* was involved in the fine tuning of ABA signaling by a feedback regulation between biosynthetic and signaling events in barley (Collin et al., 2020). Here, we cloned the *HvbZIP21* from the drought-tolerant wild barley EC_S1 and found that it was subcellularly targeted to the nucleus (Figure 6C). After overexpressing the *HvbZIP21* in *Arabidopsis*, we found that it enhanced drought tolerance in transgenic plants by increasing the activity of oxidative protective enzymes (SOD, POD and CAT) and glutathione content (Figure 7). Similar results were also found in *Poncirus trifoliata* that where the overexpression of the *bZIP* transcription factor *PtrABF*, resulted in a positive modulation of drought tolerance by scavenging ROS (Huang, 2010). Moreover, the overexpression of the *bZIP* family gene *AREB1* in *Arachis hypogaea* enhances drought tolerance by modulating ROS scavenging (Li et al., 2013). Finally, we found that silencing of *HvbZIP21* significantly restrained drought tolerance and decreased SOD, POD and CAT activities and glutathione content (Figure 8). In this study, a large number of DEGs, especially 30 hub genes, revealed in this study are potential genes that may play roles in drought tolerance. As a case, *HvbZIP21* is verified to improve drought tolerance, which doesn’t mean that *HvbZIP21* is the only reason why wild barley is more tolerant to drought stress than cultivated barley.

Taken together, the results of this study highlight the excellent gene resources found in wild barley and reveal the available genes for breeding barley with improved drought tolerance. Our study not only identified an effective drought tolerance gene but also identified 29 additional drought-related hub genes, although further experiments should be carried out to verify the function of these candidate genes. Future work will focus on understanding how *HvbZIP21* is induced and how to regulate downstream genes to manipulate ROS metabolism under drought stress.

## Data Availability Statement

The datasets presented in this study can be found in online repositories. The names of the repository/repositories and accession number(s) can be found below: National Center for Biotechnology Information (NCBI) BioProject database under accession number PRJNA828098.

## Author Contributions

WZ and CL conceived and designed the experiments. RP, SB, ZF, and LX performed all the experiments. WZ and RP analyzed and interpreted the data. RP, WZ, and CL supervised and completed the writing. All authors approved the final version of the article.

## Conflict of Interest

The authors declare that the research was conducted in the absence of any commercial or financial relationships that could be construed as a potential conflict of interest.

## Publisher’s Note

All claims expressed in this article are solely those of the authors and do not necessarily represent those of their affiliated organizations, or those of the publisher, the editors and the reviewers. Any product that may be evaluated in this article, or claim that may be made by its manufacturer, is not guaranteed or endorsed by the publisher.

## Footnotes

1
http://ftp.ensemblgenomes.org/pub/plants/release-52/fasta/hordeum_vulgare/

2
https://www.UniProt.org/
