# Supplementary material for: HvbZIP21, a Novel Transcription Factor From Wild Barley Confers Drought Tolerance by Modulating ROS Scavenging
Source: Front Plant Sci. 2022 Apr 22;13:878459. doi: 10.3389/fpls.2022.878459 (PMC9074790; doi:10.3389/fpls.2022.878459)
Supplement: Supplementary file 1 [file Data_Sheet_1.PDF]

Supplementary Material

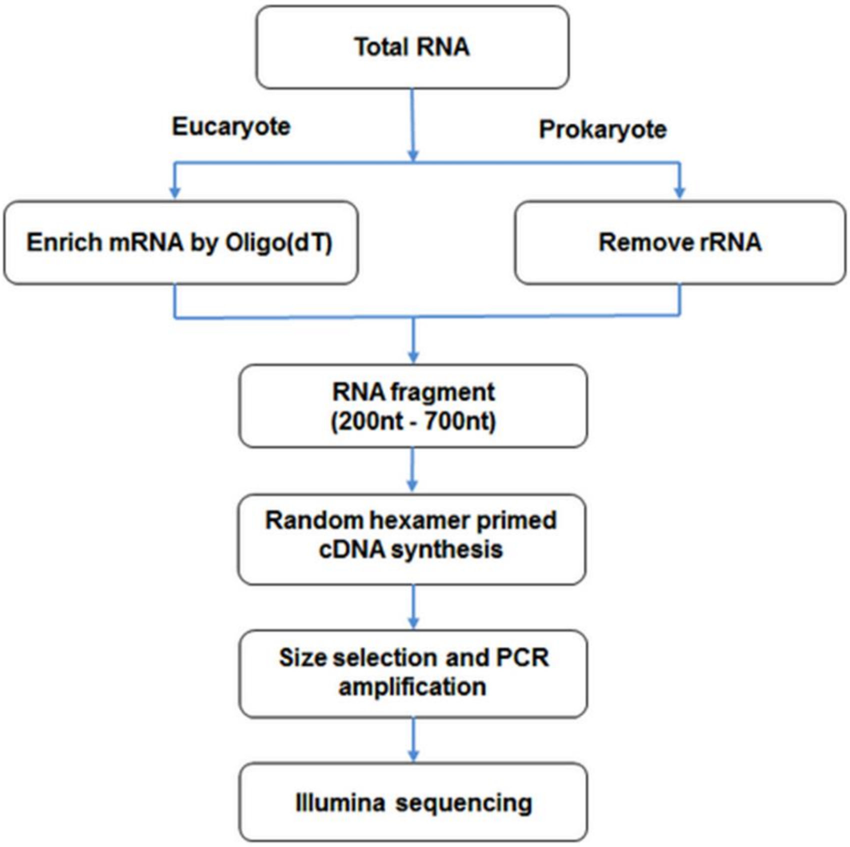

Supplementary Figure 1. RNA library construction and sequencing process.

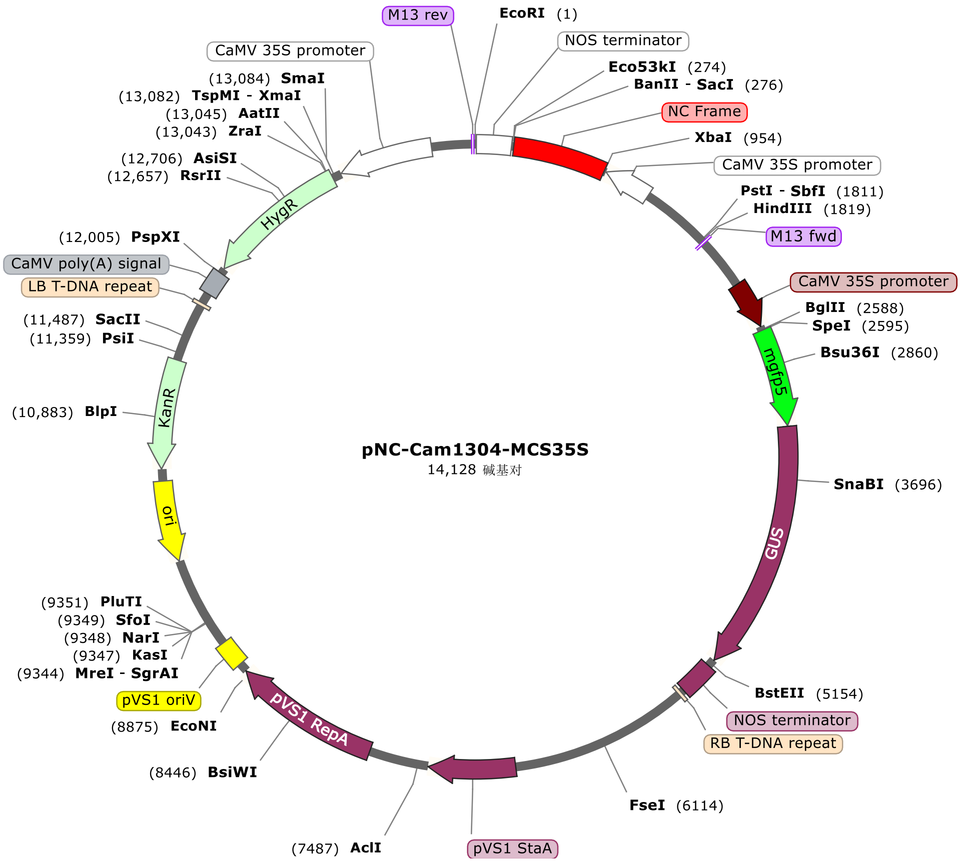

Supplementary Figure 2. The overexpression vector used in this study.

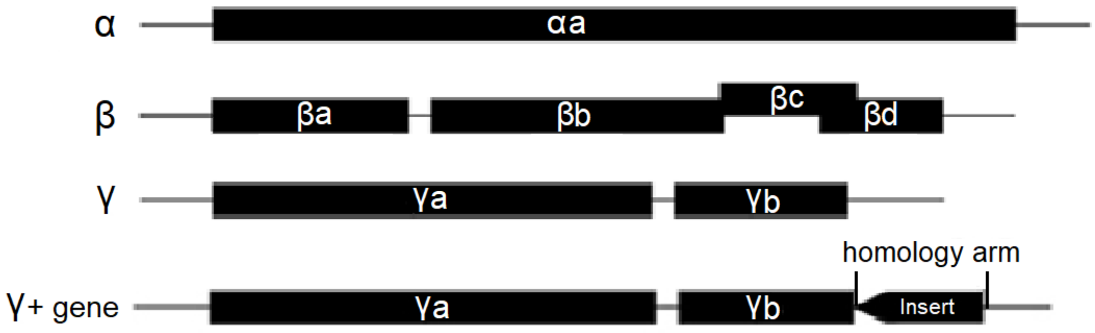

Supplementary Figure 3. The BSMV-VIGS vector used in this study.

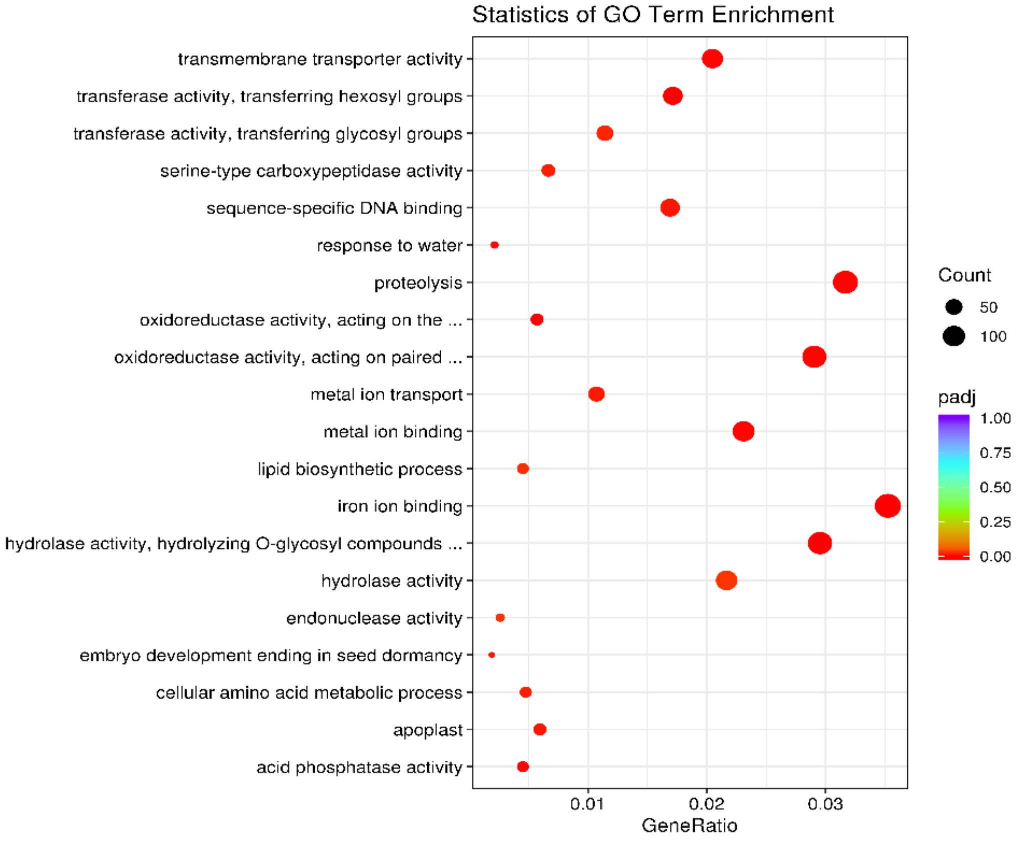

Supplementary Figure 4. GO annotation of DEGs in Baudin plants.

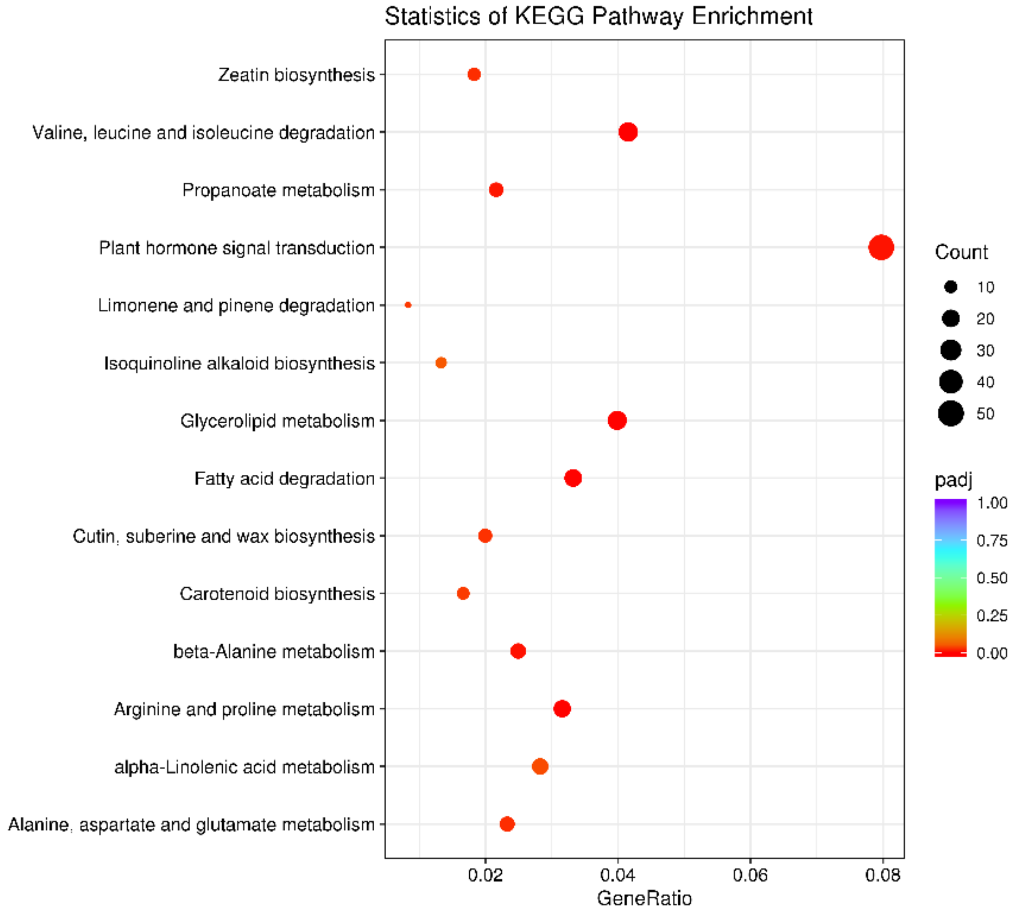

Supplementary Figure 5. KEGG enrichment of DEGs in Baudin plants.

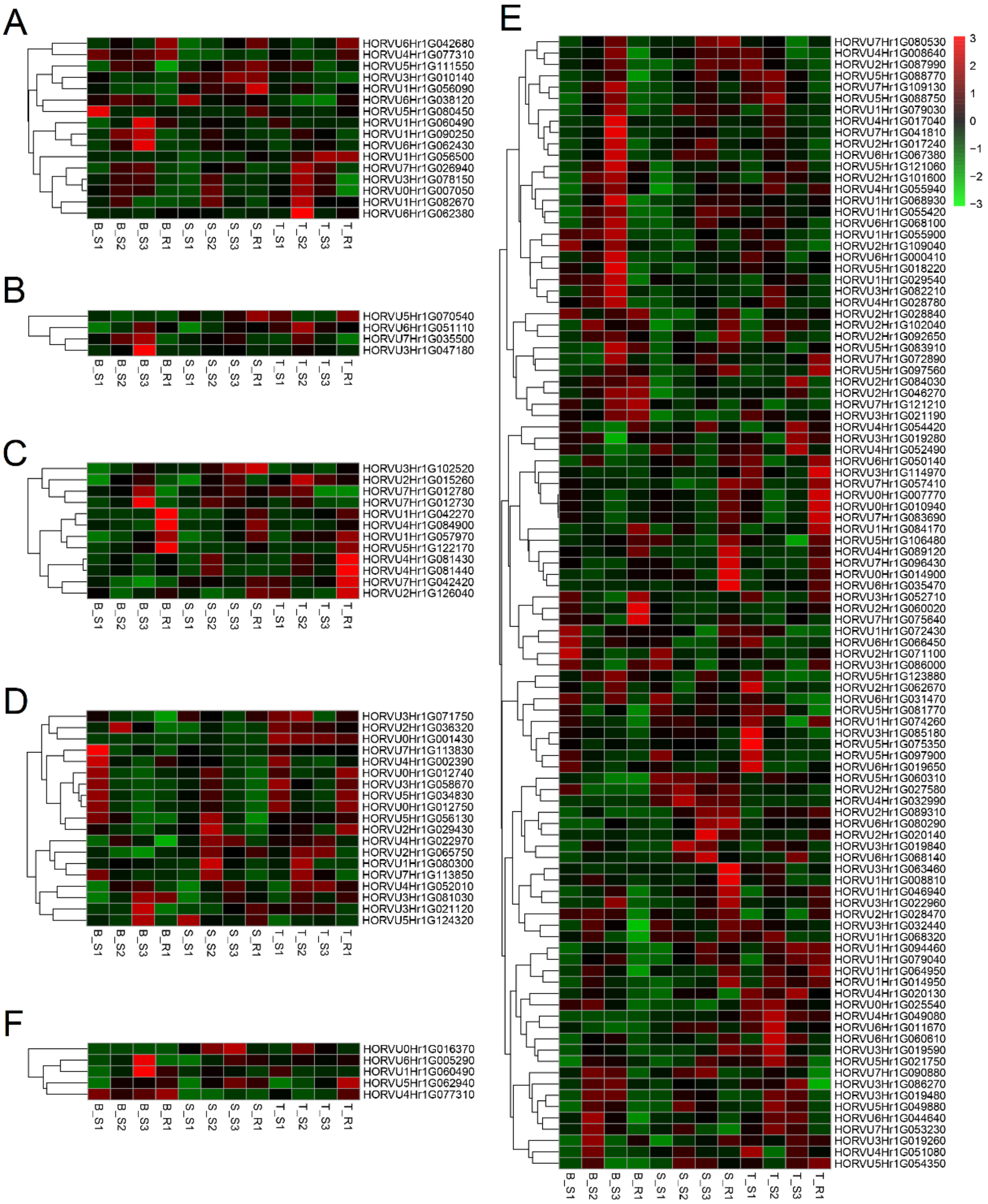

Supplementary Figure 6. Expression pattern of transcription factors under drought stress. (a) *AP2* TFs; (b) *bZIP* TFs; (c) *NAC* TFs; (d) *WRKY* TFs; (e) *MYB* TFs; (f) *DREB* TFs.

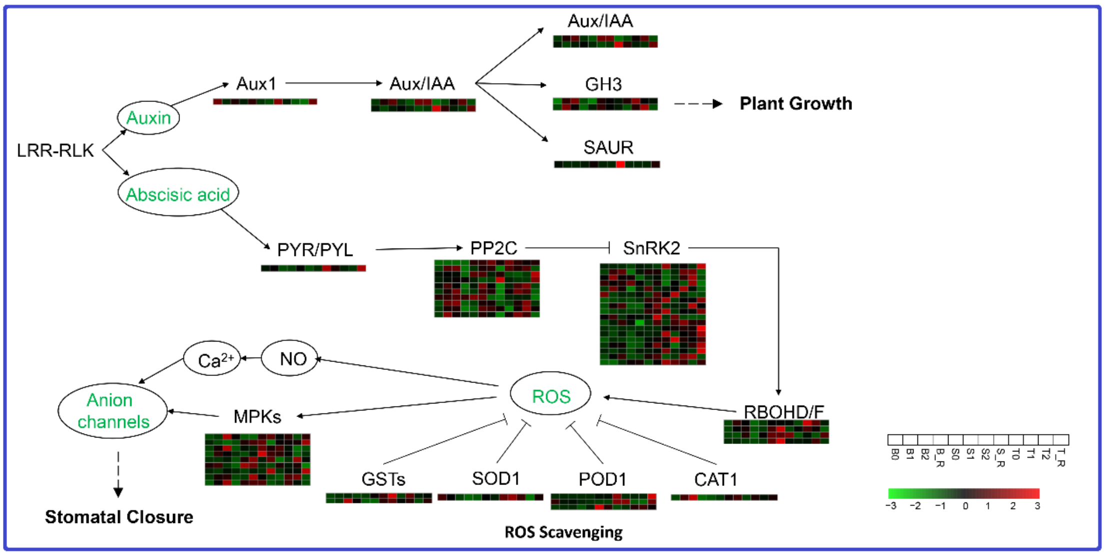

Supplementary Figure 7. The expression levels of genes in known pathways of the drought response.

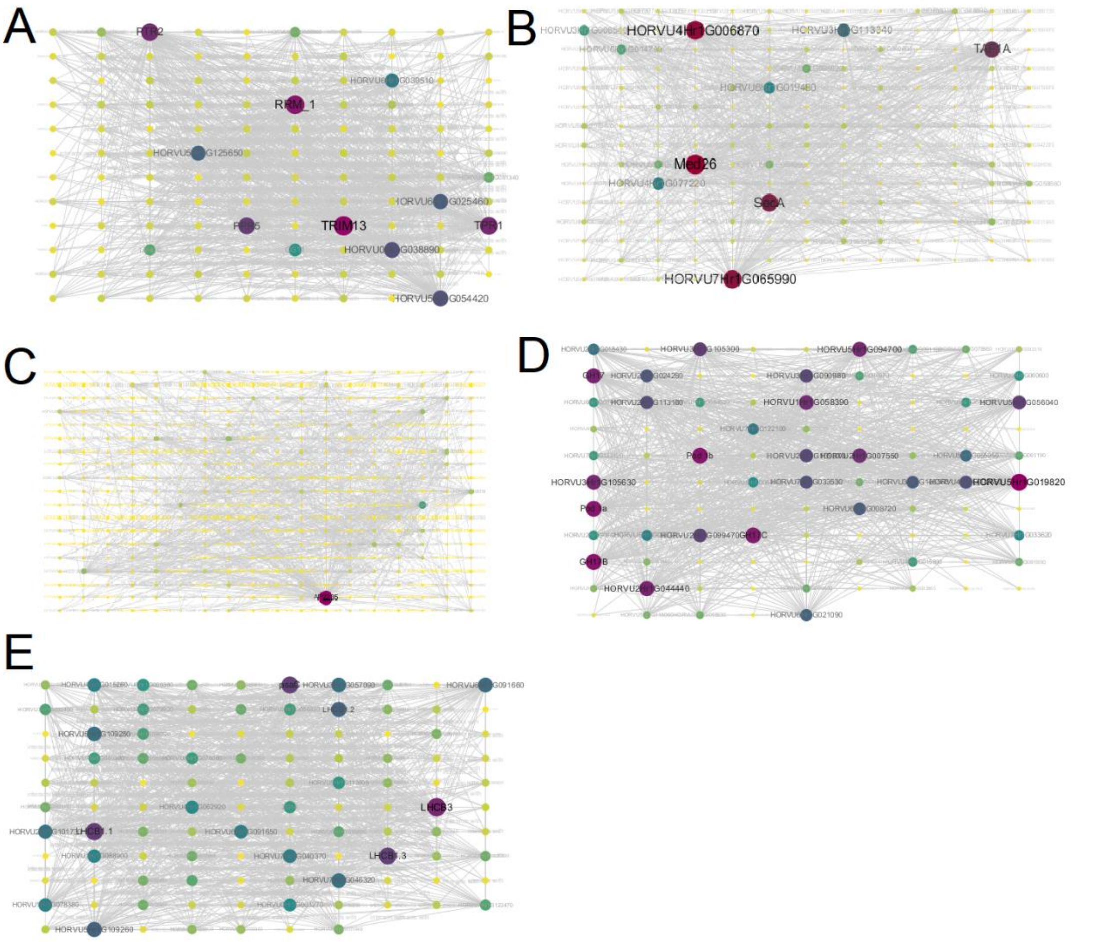

Supplementary Figure 8. The networks of 5 drought-related modules. (a) bisque4 module; (b) blue module; (c) light steel blue module; (d) steel blue module; (e) tan module.

HvbZIP21:

CTGGTCATTTTCACTTTATGAAGTATATAGCCTTTAGTCAAAAATATCCAGTTTCATTGTACT
CCTTGGTTCATCCCTTTATGTATGCCAGTAATAAAACTGAGTAAAAGAAGTAAATGTGAGCA
AACAACAAATTAAGTTGCAATTTAGTCAAGAATGGGCTGAGGCACGCGGCGCTTTCGGCAT
GCTAGTTAATATTATAGAGTTAGAGTTGTATGTTCTGGTTAGCTTGTGCTTGCTAATGACTCG
CAATTTATTTTGCTATTCTTTCTCACTCGCACGCTGATATGTGGGACACACACATCTACTGCA
TGCGTCTGGTCACCGCGCATATAGCCAGGACCGTATAGAAGCATTTTACAAGTTCAAGGAC
CATTGTGTTACGAGCAAACTAATACGAGGACCGTATGTGAAATTTACTCTTTAGTTGGCTTT
GGGGTTTTGAGCCTTCTCCGTTGTTTGCCTCTCGCTTTCTCCATCTGCTTTTGGTGCCTCTTTT
TCTCACCGCCAGCGCATCGCATCGCATCGCACCACGCCACGCCTTTGGCTTTGGTTCTTCCT
CCCACCCGCCCACTTAACTCCTAAGCAAACGCTCCTCCTCCTCGCCTATAAATTGCGTCCCT
CATCCAAGCCCCAATTCCCTTTTCCCTCTTTCTCTTCTCCCCTCTCTGGAAGGACTCGGATCC
CTTCTTTTCTTGCACCAGCAACGCCACGCTGCTGCTGCTGTTCCTTCCTATTGACCACAACTA
CCCTACCCAGCTCTTCTCTCACCTTTCAAGTTTGAACCCTCTTTGCTTGGCATCTACACCGTC
TTTAGTTCCCTGTGCCTTGAAGATTCTGTCTGAAAGTATCTCGTCATAGAAGATCTACCATCT
AGCTTAGTACTCTGCTGAAACCCTTGGCCTATGCTATGTGTTCAGGGTGACGCTCTGCTGAA
ATTTCCTGTCAGAAACCTACCTCCTTACTCAGGGCGTCACTCTGCCCAAATTTCCAGTGAGA
AACCTCCTTGTTCAGCTGCCCAAATTTCCTCTCGGAAATCAAATATTTTCACTAAGAATTCTC
GCTTGGTCAGGGAGAAACTCTGTCCGATTTCACAGTAAGAAACCTTGGTTTTTGAGGGGAG
ACTCTGCCGAATTTTTTCTATTGACCAGCTCTGCTTGTAGTTGTATGCGGCTGCTTTTATGAT
AATATCCACCGTCCTCGTCCAGTCCTTCTCCGTCGTCTTCCTCTACTGGTTCTACGTCTTCTC
ATAATCAACCAACCAACCCCGGTTAAACCAGGCTTGTTACACTACTATATTGTTTTTAATATT
TGTTGGTATAAGAAACCAAGTAGTAAAGTAATCAAGTGGTGCAAGGAAATGGCCTCTCCCG
GTCCCGGCGCGGCGTCTACCGTCATGTCGTCATCGGCCGGCTCGGAGGCGACACGGGCGC
CGTCGGCGGGGCTGATGGAGGAGAGGAAGCGCAAGCGCAAGGAGTCGAACCGGCTATCC
GCGCAGCGGTCGCGCGCGCGCAAGCTGCTGCAGGTGGACGAGCTGGAGGCGGAGGCGG
CGGCGCTGGGCGCCAAGAACTCCGCCGTGGCAGCAAAGGCGCGGGAGGCTGCGCGCCG
GTGCGCGGTCGTCCAGGCCGAGAACGAGCTCCTGCATGCTCGGGCGCTGGAGCTGGGCG
CGCGCTTGGAGTCGCTCGCCGAGTTCATCCAGTACATGGACGCAGCCGCCGACTCGGGTG
CCTCCTCCAGCCCGTTTGCCGGCGTCAGCGGCACCGCCTTGCTCCAGCAGCCGCTCCTCCA
AACGGACTTGTATTGCAATTGCAACTACACCTACTACTAGCAGATCGCATCTACTAGCTCTA
TGTTGTCTACTACTCCTTACTTGGGAGTACTAGGCATCTAGTATGTGCTTATGCTTTCCTCCC
AAGTAAGTAAGCAGTAGTGTTATTGGGTGGGCAGGAGCATGGAAATTAAAAGAGTGTTTGT
TGTAAGTGTGTCGACTGACTTTATATAGCAACTCCAACCTCCAACCGCGTGACCCAAATACT
ACATGCTTTATTTGTTGGAGCGTGCTACGCATGTTTAAAAAATATCCACCAGCTCGCGAGCC
GTTAGATCTGTCACATCAAGCGACAGAACAACTTCTTCACTTTTGCAACTTAGGTTCAGTTG
CAGTTTTTCTGTAACTATGGTTTTGTTGCAGATTTATTTTCAACAAAGATCTTATTGTAATTTTT
TTTGCAACTGAAGTTCTGTTGCAGTTTTTTTTAATTGGGATTATGTTACAGAAGCGTCGCGT
CGTTGTATTATCTAATTTTAAGGATACAGATCTTGCAACAGAGACGTTGTTGCGATAAGAAA
TTGCAACAACATGTTAGTTGCAAAAACTTTTGATGGTGATCATGAGGCACGTGACGTATAAA
GAAG

Supplementary Figure 9. The sequence of *HvbZIP21* in EC_S1. The underlined region is the coding region.

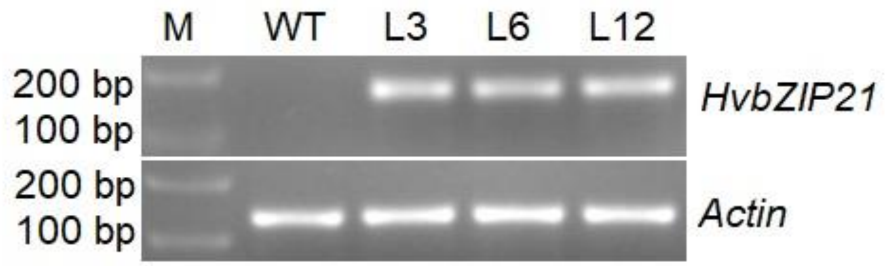

Supplementary Figure 10. The expression of the *HvbZIP21* in transgenic *Arabidopsis.*

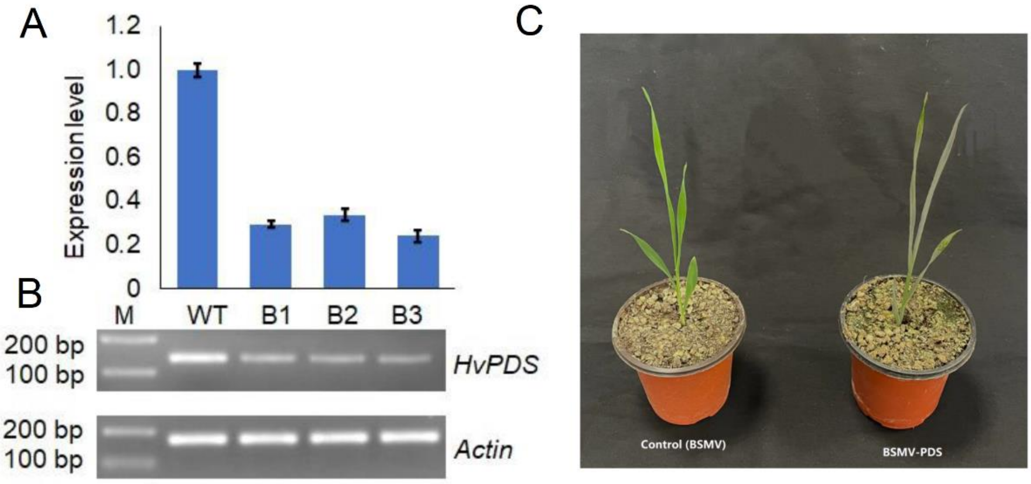

Supplementary Figure 11. The expression level of the *PDS* gene in EC_S1- and BSMV:*PDS*-inoculated plants. (a) RT-qPCR; (b) semiquantitative PCR; (c) phenotype of plants silenced with the *PDS* gene.

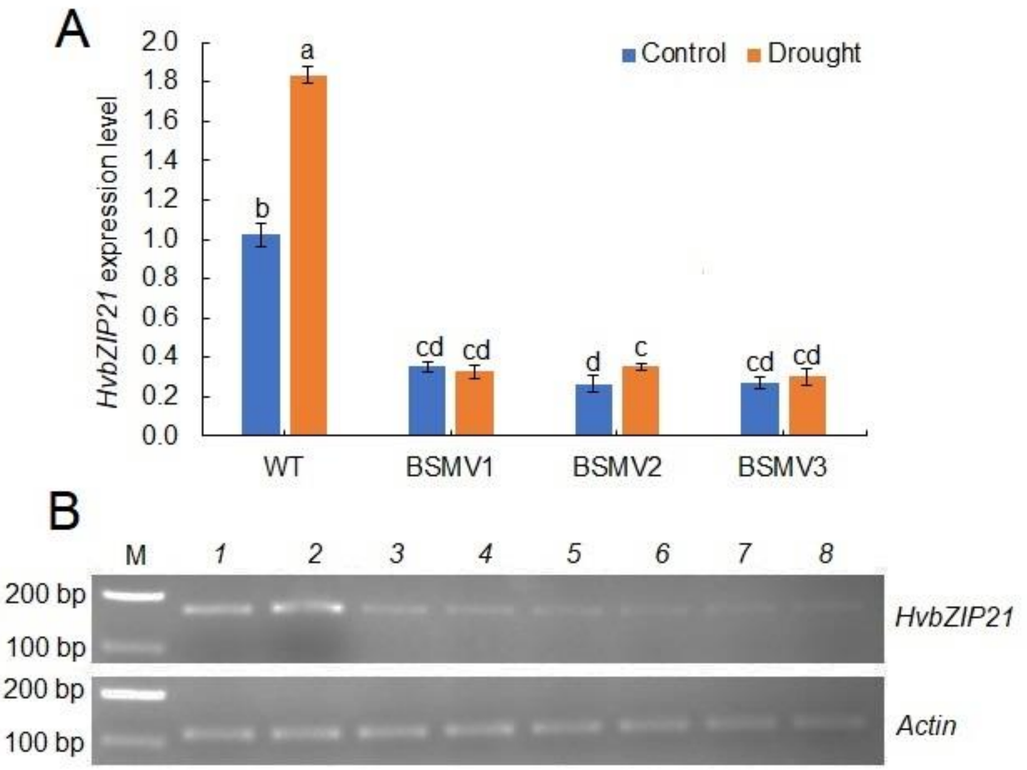

Supplementary Figure 12. The expression level of *HvbZIP21* in mock and silenced plants. (a) RT–qPCR. Different lowercase letters indicate a significant difference at $P < 0.05$. (b) The bands of PCR production. 1: BSMV:γ under well-watered conditions; 2: BSMV:γ under drought stress; 3, 5, 7: BSMV:*HvbZIP21* under well-watered conditions; 4, 6, 8: BSMV:*HvbZIP21* under drought stress conditions.
